# Distinct DNA methylation signatures in maternal blood reveal unique immune cell shifts in preeclampsia and the pregnancy-postpartum transition

**DOI:** 10.1371/journal.pone.0343041

**Published:** 2026-02-25

**Authors:** Laiba Jamshed, Keaton W. Smith, Samantha L. Wilson

**Affiliations:** 1 Department of Obstetrics & Gynecology, McMaster University, Hamilton, Ontario, Canada; 2 Department of Biochemistry & Biomedical Sciences, McMaster University, Hamilton, Ontario, Canada; Universita Politecnica delle Marche, ITALY

## Abstract

Preeclampsia (PE) is a hypertensive disorder of pregnancy characterized by immune dysregulation and significant risks to maternal and fetal health. While current management relies on high-risk patient monitoring and early diagnosis, these methods are costly and burdensome, especially for low-risk pregnancies. DNA methylation (DNAm) is a type of chemical modification that influences gene expression and has been associated with immune cell dynamics and PE pathogenesis. This study explores whether DNAm-based immune cell composition profiling can provide insights into immune dysregulation associated with PE. By also examining changes in immune cell composition across gestational timepoints and into the postpartum period, we aimed to establish a baseline of healthy immune adaptation during pregnancy, against which PE-related disruptions can be better understood. We conducted a search in the Gene Expression Omnibus (GEO) for DNAm datasets using Illumina 27K, 450K, and EPIC arrays from maternal blood in both healthy and PE pregnancies. We found two studies (GSE37722 and GSE192918) that met our criteria, involving a total of 24 healthy pregnancies and 14 with PE. To estimate immune cell composition (CD8 + T cells, CD4 + T cells, monocytes, granulocytes, natural killer cells, and B cells) from DNAm data, we applied the deconvolution algorithm developed by Houseman et al (2012). A linear model was used to assess statistical differences in immune cell proportions between PE cases and controls. Longitudinal analyses were also conducted to examine immune cell shifts during pregnancy and postpartum. No significant differences were observed between PE and control groups in any immune cell type. However, longitudinal analyses revealed substantial immune remodeling in the postpartum period, characterized by decreased monocytes and granulocytes, and increased natural killer cells, B cells, and T cells. While subgroup analyses showed some variability in significance, particularly in GSE192918, the overall trends were consistent across datasets, emphasizing the importance of gestational age in immune dynamics. These findings support the use of DNAm profiling as a valuable tool for characterizing immune cell dynamics during pregnancy. Although immune differences between PE cases and controls were not observed with the Houseman method, longitudinal shifts were consistently captured and provide additional insights into the evolution of immune changes from pregnancy to postpartum, supporting the potential of DNAm-based profiling for developing predictive and monitoring tools for pregnancy and pregnancy-related pathology. It is important to note that these analyses were based on a single deconvolution approach applied to a cohort with well-matched clinical criteria; and that differences in study design, timing of sample collection, and cohort characteristics may limit broader generalizability. Future studies leveraging pregnancy-included reference matrices in deconvolution methods and larger, more diverse cohorts are essential to refine the application of DNAm-based immune profiling in pregnancy and pregnancy complications.

## 1. Introduction

### 1.1. Overview of Preeclampsia

Preeclampsia (PE) is a pregnancy-specific disorder characterized by new-onset hypertension and proteinuria after 20 weeks of gestation.[1] Occurring in an estimated 3–5% of pregnancies, PE is a leading cause of both maternal and fetal morbidity and mortality worldwide.[2] Although PE is a common complication of pregnancy, its exact etiology and pathogenesis are not fully understood, making early detection and intervention challenging. As a multisystemic disorder, the pathogenesis of PE has been associated with poor placentation, placental hypoxia, endothelial dysfunction, impaired angiogenesis, and excessive maternal inflammation.[2,3] Together, these altered pathways ultimately lead to a placental ischemic microenvironment, inadequate uterine vascular remodeling, compromised blood perfusion and oxidative stress.[2–5] Collectively, these factors contribute to the hallmark clinical features of hypertension and organ dysfunction including renal, hepatic, hematological or neurological complications.[2–5] If left untreated, PE can lead to severe maternal complications, including cerebral hemorrhage, multi-organ failure (i.e., liver rupture, myocardial infarction, kidney failure), placental abruption, and heart disease later in life.[6–9] Infants from preeclamptic pregnancies are at increased risk for intrauterine growth restriction (IUGR), fetal death, and premature delivery.[7] While the definitive treatment for PE remains the timely delivery of the placenta and the baby, clinicians must carefully balance the urgent maternal need for delivery against the developmental benefits of prolonging the pregnancy for the fetus. Early identification of pregnancies at risk of PE enables tailored clinical management strategies [10], including the initiation of low-dose aspirin before 16 weeks and enhanced monitoring, ultimately improving maternal and fetal outcomes.[11] Recent advances in prenatal care, such as risk prediction models, diagnostic tests measuring angiogenic factors [12–15], and the adoption of home monitoring technologies [16–18], have significantly contributed to the management of PE. Despite these advancements, the lack of standardized and non-invasive testing continues to hinder the early identification of pregnancies at risk of PE. As such, there is an urgent need for consistent screening protocols to improve maternal and fetal outcomes.

### 1.2. Immune system adaptations in pregnancy and preeclampsia

Pregnancy induces significant immune composition changes, promoting maternal tolerance and fetal development.[19] In normotensive pregnancies, regulatory adaptations at the maternal-fetal interface involving immune cell function and immune cell numbers, promote maternal tolerance to the developing fetus, support fetal growth and maintain maternal health (Reviewed in: [19]). These changes include increased regulatory T cells (Tregs) recruited from maternal peripheral blood to the fetal-maternal interface, and a shift towards anti-inflammatory macrophages.[20] However, in PE, these adaptions are disrupted, leading to a dominance of pro-inflammatory immune cells and a reduction in regulatory immune cells in peripheral blood.[20] This imbalance in immune cell populations impedes maternal immune tolerance to the semi-allogenic fetus, and is thought to contribute to the pathogenesis of PE (Reviewed In: [2,21,22]). Monocytes, as precursors of tissue macrophages, are integral to the innate immune response, responsible for maintaining tissue integrity and responding to pathogens. In preeclamptic pregnancies, however, monocytes exhibit altered phenotype and functions, which contribute to the systemic inflammatory state characteristic of PE. Specifically, pro-inflammatory M1 macrophage activation increases, compounding the inflammatory response, leading to endothelial dysfunction and systemic inflammation.

Neutrophils, typically the first responders of the immune system, also display increased activation and adhesion in PE, leading to the release of reactive oxygen species and additional endothelial damage.[23] Neutrophils serve as primary effectors in systemic inflammation by recruiting, activating, and reprogramming other immune cells, including dendritic cells, B cells, natural killer (NK) cells, CD4 and CD8 T cells, and mesenchymal stem cells. In PE, B cells also exhibit dysregulation, with altered antibody production that may further modify the placental immune environment.[22,24,25]

An imbalance in T cell subsets is another feature, characterized by an increased presence of pro-inflammatory T helper 1 (Th1) cells and a decrease in uterine and circulatory regulatory T cells (Tregs).[26] Th1 cells release inflammatory cytokines, which further amplify the systemic inflammatory response in PE, while Tregs are essential for maintaining immune tolerance and suppressing excessive inflammation. This shift in the Th1/Treg ratio exacerbates the inflammatory conditions associated with PE.[21] Moreover, a reduction in both the number and activity of NK is observed in PE.[27] NK cells are crucial for immune surveillance and tolerance at the maternal-fetal interface, yet in PE, they display functional dysregulation within both maternal circulation and placental tissues. Collectively, these disruptions in the regulation of innate and adaptive immune cells and their cellular responses create a cytotoxic *in utero* environment, intensifying the pathological landscape of PE.[28]

### 1.3. Current Immune Markers and Limitations

In recent years, there has been a surge in research aimed at identifying predictive markers of PE, focusing on systemic inflammatory markers and immune cell signatures.[12,28–34] Markers such as the neutrophil-to-lymphocyte ratio [29–31] (NLR) and monocyte-to-lymphocyte ratio [35] (MLR) are elevated in PE, associated with increased oxidative stress, endothelial dysfunction, and vascular damage. While promising, the utility of these immune cell ratios as a screening tool for PE are limited by sensitivity [34], patient population applicability and comorbidities [32,33,36], and predictive value for disease severity, restricting their effectiveness as screening tools.

### 1.4. Placental interactions and immune cell function

The complete extent of immune system dysregulation in PE is still not fully understood. The specific inflammatory pathways involved and the dynamic interactions between immune cells and other placental cells (e.g., trophoblasts and stromal cells) require further exploration. This complex interplay between immune cells and their interaction with placental cells likely forms a distinct cellular signature that may provide insights into the pathophysiology of PE and open avenues for potential diagnostic strategies. As trophoblasts play an essential role in early pregnancy by facilitating blastocyst attachment, invasion, migration, and endometrial remodelling, they are particularly susceptible to disruptions in the immunological microenvironment. Starting at 8 weeks of gestation, endovascular trophoblasts begin remodelling the spiral uterine arteries. The effectiveness of this placentation process is dependent on the ability of the extravillous trophoblasts to avoid detection by the maternal immune response.[37–39] If maternal tolerance towards fetal components diminishes during this period, shallow placentation may occur, a condition frequently associated with PE.[40,41]

As pregnancy progresses, increased immune responses may exacerbate placental inflammation and lead to widespread systemic inflammation in the pregnant individual. Excessive activation of the maternal immune system can stimulate the release of proinflammatory cytokines and antiangiogenic factors within the fetoplacental unit and vascular endothelium.[42] These molecules are critical mediators of intercellular communication but, in this context, amplify the immune response, further exacerbating systemic inflammation. The overactivation of maternal immune cells—including monocytes, macrophages, neutrophils, T cells, NK cells, and dendritic cells—against trophoblasts has been identified as an important mechanism triggering trophoblast apoptosis and cell death.[43] This overactivity disrupts the remodelling of maternal uterine arteries, intensifying placental dysfunction. Abnormal proportions and functions of immune cells, including T cells, B cells, macrophages, and NK cells, create an immunological environment conducive to increased reactivity against trophoblasts.[21,22,39] This heightened reactivity and immune dysfunction can be identified and measured by assessing immune cell counts and composition through advanced analytical tools.

### 1.5. DNA methylation as a tool for immune composition analysis

Advanced techniques such as flow cytometry, mass cytometry (CyTOF), single-cell RNA sequencing (scRNA-seq), and immunohistochemistry (IHC) have been used to accurately characterize immune cell activity and composition.[44,45] Recently, DNAm cell deconvolution algorithms have emerged as a promising approach for identifying immune cell signatures, particularly in complex conditions like PE. Unlike traditional methods that require cell sorting, DNAm can infer immune cell proportions from bulk samples using cell-specific signatures and stable epigenetic modifications, making it especially useful in detecting subtle shifts in immune cell dynamics in PE.[46]

Most studies on DNAm changes during PE have focused on placental tissue [47] or cord blood [48], likely due to the historical emphasis on the placenta as the central driver of PE pathology and the accessibility of these tissues postpartum. However, maternal blood analysis is especially critical as it provides a direct and systemic view of immune-related changes, capturing cell-specific methylation patterns and proportions that could offer critical insights into maternal immune dynamics in PE. Reference-based algorithms [49], which use DNAm data from sorted immune cell types obtained through flow cytometry, can estimate cell type proportions from bulk samples without requiring physical separation and sorting the immune cells. Given that both immune dysregulation and altered DNAm are both linked to PE [50]—and that DNAm plays a central role in immune cell development and function [51]—these shifts in cell proportions are inherently reflected in DNAm patterns associated with PE. Analyzing DNAm profiles of whole blood allows for the estimation of immune cell proportions, helping to pinpoint specific immune changes tied to PE. With a clinical need for reliable, pre-symptomatic PE prediction in both low- and high-risk pregnancies, profiling immune cell signatures through maternal blood sampling could be valuable for identifying and monitoring PE, supporting earlier intervention and improved patient management.

In this study, we used DNAm signatures from existing datasets to identify and characterize the immune cell composition in maternal blood samples from PE patients. Additionally, given the emerging research on DNAm changes throughout gestation, we further used DNAm profiles to investigate how immune cell composition might vary across different stages of pregnancy. By correlating DNAm signatures with altered immune cell proportions, this dual approach of identifying immune shifts across a normotensive gestational timeline and comparing normotensive and PE cases, offers a non-invasive framework for future studies aimed at early immune monitoring and PE risk stratification.

## 2. Methods

### 2.1. DNA methylation sample selection and data processing

**Fig 1:**
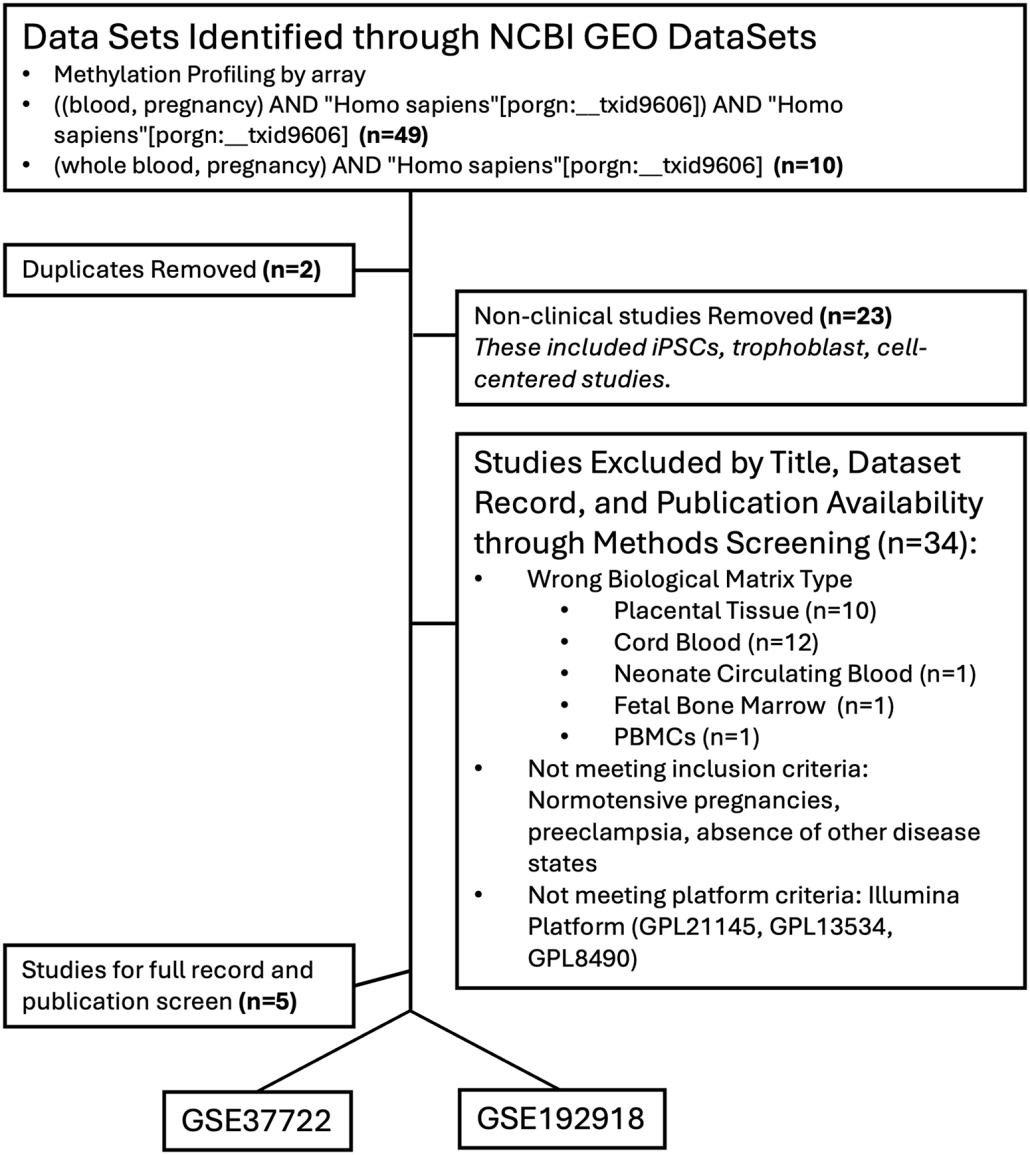
Flow chart of GEO dataset selection. The initial search yielded 59 studies. After removing two duplicates, 57 studies were screened for relevance based on the inclusion criteria, which included the use of human clinical samples (excluding human cells/stem cells/iPSCs). Further screening of the remaining 34 studies involved assessing biological matrix, normotensive pregnancies, PE, data sufficiency, absence of other disease states, and the use of the Illumina platform. Five studies met the criteria for deeper screening, of which two were selected for the final analysis.

This study analyzed DNAm datasets from maternal whole blood samples of pregnant individuals encompassing normotensive and preeclamptic pregnancies. A search query combining whole blood, pregnancy, and *Homo Sapiens* was employed, resulting in 34 studies (Fig 1). Out of the identified studies, five were further selected based on their profiling methods: Methylation profiling by array. All five studies involved samples described as either whole blood or peripheral whole blood. DNAm data were sourced from the Gene Expression Omnibus (GEO) database. To ensure high coverage, reproducibility, data quality and ease of analysis, the DNAm dataset screening was limited to the Illumina platform (i.e., Illumina 27K, 450K, and EPIC arrays). Two datasets, GSE37722 (HumanMethylation27 BeadChip arrays) and GSE192918 (Infinium MethylationEPIC BeadChip array), were selected for analysis. Both datasets were whole/peripheral blood samples. Only one study, GSE37722 included samples from normotensive (control, N = 14) and preeclamptic pregnancies (N = 14). In both datasets, the authors processed and normalized the raw data using GenomeStudio. Only the normalized data was available on GEO.

DNAm data were systematically retrieved from GEO using the GEOquery package [52] (version 2.66.0) within the R statistical environment (version 4.2.1). This procedure specifically targeted the Series Matrix files of the GSE37722 and GSE192918 datasets containing β-values, which are represent the ratio of the methylated probe intensity over the sum of methylated and unmethylated probe intensities. β-values from each dataset were normalized using quantile normalization (preprocessCore package, version 1.60.2).[53] Although quantile normalization does not fully mitigate batch effects, it ensures comparability across datasets by aligning data distributions, enhancing the reliability of biological interpretations.[54] Immune cell deconvolution was performed using an algorithm developed by Houseman et al (2012) [55] to infer the composition of blood cell mixtures from DNAm profiles.

### 2.2. Cell-deconvolution for leukocyte composition estimation

The Houseman algorithm for cell type deconvolution utilizes a reference-based approach to estimate the proportion of different cell types (monocytes, granulocytes, natural killer cells, B cells, CD4 + T cells, and CD8 + T cells) in a mixed cell population found in whole blood.[55] Houseman’s software (version 2) and cell-specific reference DNAm profiles for cell type deconvolution were obtained from the supplementary files accompanying their publication describing their algorithm. Cell-deconvolution was performed on GSE37722 and GSE192918, using the 100 most informative CpGs available in each dataset to estimate cell type proportions for each sample. The data analyzed in this study were obtained from publicly available datasets in the Gene Expression Omnibus (GEO), specifically under accession numbers GSE37722 and GSE192918. The processed data and analysis scripts used in this study are openly available in the GitHub repository at https://github.com/WilsonPregnancyLab/ImmuneCellComposition.

#### 2.2.1. Changes in immune cell composition between Normotensive and Preeclamptic Pregnancies.

The immune cell proportion data was compared between ‘Normotensive’ and ‘Preeclamptic’ pregnancies (which included only GSM identifiers from the GSE37722 dataset).

#### 2.2.2. Changes in immune cell composition across gestation.

The datasets GSE192918 and GSE37722 were both used to independently investigate immune cell composition across different stages of pregnancy. For each dataset, the series matrix was individually extracted, and sample IDs were identified and matched to specific gestational timepoints. These stages were categorized as ‘early-pregnancy’, ‘mid-pregnancy’, ‘at delivery’ and ‘postpartum’. GSE192918 defined these stages as follows: early-pregnancy (10–14 weeks), mid-pregnancy (24–28 weeks), at delivery (38–40 weeks), and postpartum (10 month post-delivery). While GSE37722 also included data for normotensive pregnancy stages (i.e., early, mid, delivery, postpartum), the exact timings of these stages were not explicitly defined in the GEO database. Subsequent processing involved quantile normalization of both datasets, application of the Houseman algorithm to estimate immune cell fractions, and stratification of these estimates by gestational timepoint.

### 2.3. Data visualization and statistics

#### 2.3.1. Comparing normotensive and preeclamptic pregnancies.

All statistical analyses were conducted within the R statistical environment (version 4.2.1). As the datasets did not meet the assumptions of normality and equal variance, the Wilcoxon rank-sum test was used to determine significant difference in cell-type proportions and DNAm levels between normotensive and preeclamptic pregnancy groups. The Kolmogorov-Smirnoff test was used to determine differences in the overall distribution of cell type composition between normotensive and preeclamptic pregnancies. A p-value of ≤ 0.05 was considered statistically significant.

#### 2.3.2. Changes in immune cell composition across gestation.

Linear modelling was used to assess changes in the proportion of immune cell subtypes at different time points across gestation in both GSE192918 and GSE37722. For each immune cell subtype, a linear model was constructed using the limma package [56] (version 3.54.2) in R (version 4.2.1). Contrasts were applied to compare the estimated fraction of each immune cell subtype between each pairwise combination of time points. A false discovery rate (FDR)-corrected p-value was calculated for each comparison. An FDR cut-off of 0.05 was used as a threshold for significance. Plots for this data were produced with the ggplot2 library (version 3.4.3) in R. Additionally, an Anderson-Darling all pairs comparison test was used to assess differences in the overall distribution of cell type proportions across timepoints. All pairwise combinations of timepoints were tested. A p-value of ≤ 0.05 was considered statistically significant.

## 3. Results

### 3.1. Immune Cell Composition does not significantly differ between Normotensive and Preeclamptic pregnancies

**Fig 2:**
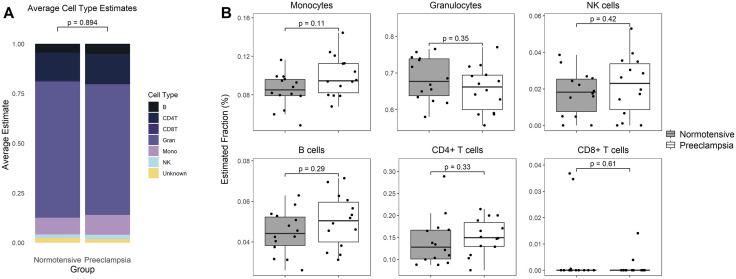
(A) Average Estimated Cell Type Distribution and (B) Immune cell proportion between normotensive (grey, control) and preeclamptic (white) pregnancies. Houseman (2012) was used to deconvolute GSE37722 (N = 14 control, N = 14 preeclamptic pregnancies) to identify changes in the proportion of monocytes, granulocytes, natural killer cells, B-cells, CD4 + T cells and CD8 + T cells. All data is presented as mean. A Kolmogorov-Smirnoff test was used to assess differences in the overall distributions, while a Wilcoxon test was conducted for each cell type. A p-value of ≤ 0.05 was considered statistically significant.

Immune cell proportion of monocytes, granulocyte, natural killer cells, B cells, and both T cell subtypes, did not exhibit statistically significant changes between normotensive and preeclamptic pregnancy groups (Fig 2), although monocytes show a modest, non-significant increase in PE pregnancies (p = 0.11).

### 3.2. Immune cell composition varies with gestational age, with the most pronounced changes occurring in the postpartum period

**Fig 3:**
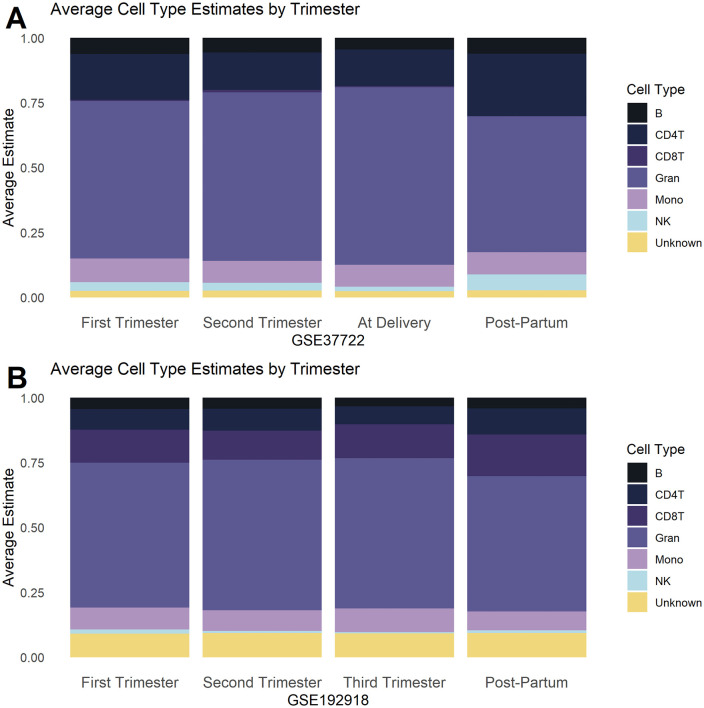
Average Estimated Cell Type Distribution across gestation for (A) GSE37722 and (B) GSE192918. Houseman (2012) was used to deconvolute both GEO sets to identify changes in the proportion of monocytes, granulocytes, natural killer cells, B-cells, CD4 + T cells and CD8 + T cells across ‘first trimester, ‘second trimester’, ‘third trimester’ or ‘at delivery’ and ‘postpartum’. While GSE37722 included data for normotensive pregnancy stages, the exact timings of these stages were not explicitly defined in the GEO database. Differences in distribution across gestational were assessed using an Anderson-Darling all pairs comparison test. A p-value of ≤ 0.05 was considered statistically significant.

**Fig 4:**
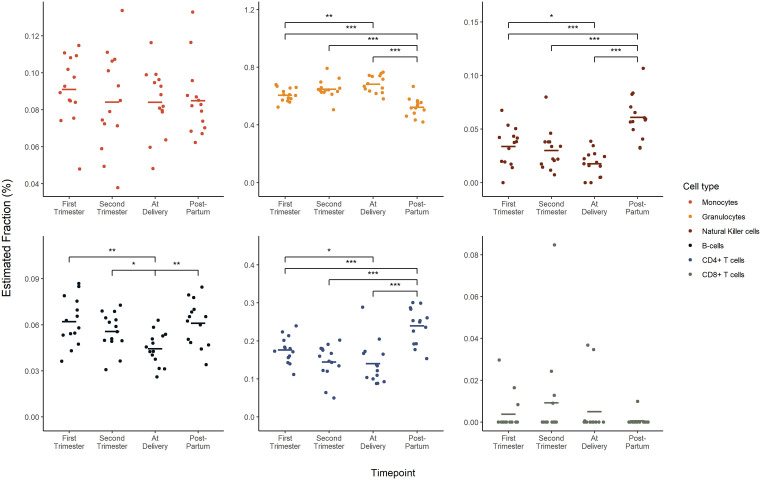
Immune cell composition across gestational age within GSE37722. Houseman (2012) was used to deconvolute both GEO sets to identify changes in the proportion of monocytes, granulocytes, natural killer cells, B-cells, CD4 + T cells and CD8 + T cells across ‘first trimester, ‘second trimester’, ‘at delivery’ and ‘postpartum’. While GSE37722 included data for normotensive pregnancy stages, the exact timings of these stages were not explicitly defined in the GEO database. All data is presented as mean. A linear model was conducted for each cell type. An FDR-adjusted p-value of ≤ 0.05 was considered statistically significant.

**Fig 5:**
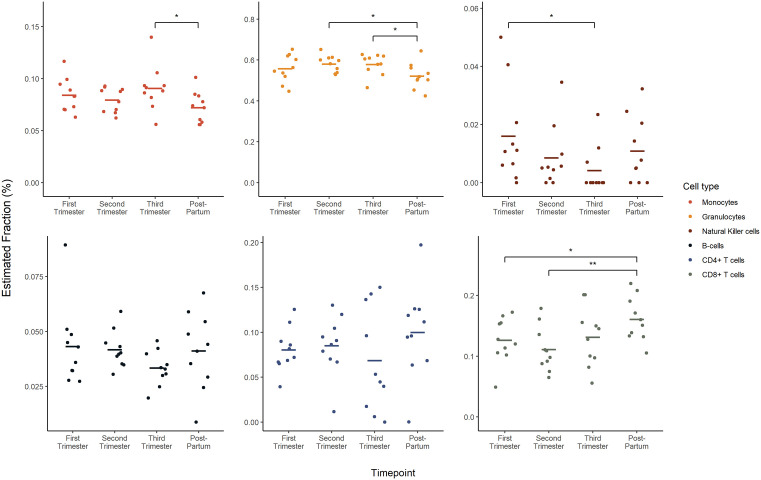
Immune cell composition across gestational age within GSE192918. Houseman (2012) was used to deconvolute both GEO sets to identify changes in the proportion of monocytes, granulocytes, natural killer cells, B-cells, CD4 + T cells and CD8 + T cells across ‘first trimester, ‘second trimester’, ‘third trimester’ and ‘postpartum’. GSE192918 defined these stages as follows: early-pregnancy (10-14 weeks), mid-pregnancy (24-28 weeks), at delivery (38-40 weeks), and postpartum (10 month post-delivery). All data are presented is mean ± SEM. A linear model was conducted for each cell type. An FDR-adjusted p-value of ≤ 0.05 was considered statistically significant.

In our analysis of the gestational age across datasets, significant changes were observed in the immune cell profiles between the delivery and postpartum periods (Figs 3–5). Although there were no significant differences between trimesters in either dataset, the greatest changes in immune cell profiles were observed postpartum.

In dataset GSE37722, where blood samples at delivery were collected within a 24 h window of birth, significant changes in immune cell profiles (granulocytes, NK cells, B cells and CD4 + T cells) were observed between the delivery and postpartum periods (Fig 4). Specifically, granulocyte proportions decreased after delivery, while natural killer cells, B cells, and CD4 ⁺ T cells increased. Interestingly, during pregnancy, granulocytes showed an increasing trend from the first trimester through delivery, whereas NK cells, B cells, and CD4 ⁺ T cells decreased over the same period. Significant shifts in granulocytes, NK cells, B cells, and CD4 + T cells were also detected between the first and second trimesters and the postpartum period, as well as between the first trimester and delivery. In contrast, monocytes and CD8 ⁺ T cells remained relatively stable across all timepoints.

In our analysis of the GSE192918 dataset, similar to GSE37722, significant changes in immune cell profiles were observed between the third trimester and the postpartum period. Notably, there was a significant reduction in monocytes and granulocytes, accompanied by an increase in CD8 + T cells. While similar trends were observed, specifically increasing granulocyte proportions during pregnancy followed by a reduction postpartum, and decreasing NK cells, B cells, and CD4 + T cells during pregnancy followed by postpartum increases, these changes were not statistically significant in GSE192918. There were no significant changes in B cells or CD4 + T cells across gestation or in the postpartum period.

## 4. Discussion

### 4.1. Immune cell composition in Preeclampsia: Quantitative versus Functional changes

Numerous studies have explored the relationship between leukocyte counts and PE to better understand the underlying immune dysregulation (Reviewed in: [29]). However, total leukocyte counts and basic differential compositions (e.g., neutrophils vs. lymphocytes) provide a readily accessible, but limited view into functional status or cellular activation state of immune cells in PE. In this study, we present a proof of concept that DNAm-based immune cell deconvolution can estimate leukocyte composition in maternal blood during pregnancy, offering an efficient alternative to more labor-intensive techniques like flow cytometry. We did not observe significant differences in granulocyte, B cell, NK cell, or CD4 + /CD8 + T cell proportions between normotensive and preeclamptic pregnancies. Although we did not detect shifts in relative abundance, prior research has established immune dysregulation as a hallmark PE, involving both innate and adaptive immune cells and their associated signaling pathways (Reviewed In: [22,57]). NK cells promote vascular remodeling of the uterine spiral arteries, while CD4 + T cells maintain immune tolerance and pathogen defense. Chronic activation of T cells, B cells, macrophages, and NK cells contributes to placental ischemia and systemic inflammation. B cells also produce anti-angiotensin II type 1 receptor autoantibodies, which impair endothelial function, and neutrophils mediate oxidative stress and tissue damage. Monocytes, although not significantly altered in our dataset (p = 0.11), exhibited a modest trend toward increased proportions in preeclamptic compared to normotensive pregnancies. While this difference did not reach statistical significance, it aligns with observations that monocyte behaviour in PE may involve functional dysregulation rather than substantial changes in abundance.[58–62] Monocytes are key players in the innate immune system and contribute to tissue remodeling and immune surveillance through their differentiation into macrophages and dendritic cells. [63,64] During healthy pregnancy, monocyte activation increases with gestational age, alongside elevated granulocyte levels and pro-inflammatory cytokines such as IL1β, IL6, and IL8.[59,60] In PE, monocytes display dysregulated phenotypes, including heightened activation, reduced CD14 expression, impaired vascular adherence, and diminished immunosuppressive capacity.[43,58] Several studies have reported unchanged monocyte counts between normotensive and preeclamptic individuals, further supporting the idea that qualitative shifts – rather than quantitative ones – underlie PE-related immune dysfunction.[58,61,62,65] Some studies have proposed that the monocyte-to-lymphocyte ratio may serve as a more sensitive indicator of disease severity [35,36,66,67], however, findings remain inconsistent across cohorts. This variability, potentially stemming from differing PE diagnostic criteria (e.g., ACOG vs ISSHP) [6,68] and patient heterogeneity [69], emphasizes the need for complementary, mechanism-focused approaches to assess immune dysfunction in PE.

### 4.2. Methodological considerations in immune cell estimation

As we detected no statistically significant difference in immune-cell proportions between preeclamptic and normotensive pregnancies; this outcome likely reflects both biological complexity, clinical heterogeneity, and methodological constraints. The relatively small sample size may have limited out power to capture subtle shifts and current DNAm deconvolution provides only bulk estimates that cannot resolve subset-specific or activation-state changes. Comparatively, while studies investigating absolute immune cell counts [58,61,62] typically use flow cytometry to directly evaluate cell surface markers, our study applies a DNAm-based deconvolution method [55] to computationally infer immune cell proportions from bulk blood samples. We used the Houseman algorithm, which leverages cell-type-specific methylation signatures to estimate leukocyte composition from bulk blood samples. Flow cytometry provides high-resolution data on cell surface markers and immune cell subsets but requires fresh samples, is labor-intensive, and is limited in the number of markers that can be analyze markers simultaneously. In contrast, DNAm-based deconvolution offers a scalable, non-invasive approach to estimate immune cell proportions by detecting unique, cell-specific epigenetic signatures. These signatures provide insights into immune composition that are not solely dependent on surface antigen expression, thus allowing for broader cell-type identification in bulk, complex, or archived tissue samples.[70]

In this analysis, we applied the Houseman reference matrix [55], to estimate immune cell proportions from DNAm data. This approach relies on a set of ~100 CpG sites that are differentially methylated between leukocyte subtypes, measured using the Illumina Human Methylation arrays. The algorithm applies a reference matrix generated from fluorescence-activated cell sorting (FACS)-purified leukocyte subgroups, derived from 46 purified white blood cell samples and 27 whole blood controls to account for batch effects. Although this method provides a robust framework for estimating immune composition, and has been validated for estimating major leukocyte subset, it is important to acknowledge that the reference was developed from non-pregnant adult donors, and the biological sex of these donors was not explicitly reported. This lack of sex-specific information limits our ability to assess how well the reference reflects sex-based immunological differences and hormonal differences which are critical in pregnancy and pregnancy-related conditions. [71–73] However, it is important to emphasize that, despite these limitations, our results remain robust. Both the normotensive and PE groups were analyzed using the same reference matrix and algorithm, so any biases introduced by this reference would affect both groups equally, preserving the validity of relative comparisons.

Future work would benefit from developing pregnancy- and sex-specific reference panels to enhance the biological relevance and sensitivity of immune deconvolution in maternal health research. As highlighted by Barton et al. (2019), incorporating Houseman-derived cell-type estimates as covariates can introduce multicollinearity, resulting in unstable regression coefficients and inflated standard errors.[74] In their analysis of the Raine Study, a longitudinal pregnancy cohort, they found that adjusting for estimated immune cell proportions altered both the direction and significance of associations in methylation models. These findings highlight the importance of applying caution when incorporating DNAm-based deconvolution outputs into regression analyses, particularly in pregnancy datasets where immune variation is both dynamic and tightly linked to physiological outcomes.

In addition to statistical concerns, biological limitations of DNAm deconvolution should also be considered, particularly its reliance on predefined reference matrices derived from correlating DNAm data with flow cytometry data. Although flow cytometry offers detailed resolution of immune subsets, including classical (CD14^+^CD16^−^) and non-classical (CD14^low^CD16^+^) monocytes, as well as monocyte-derivatives such as dendritic cells and macrophages, most DNAm reference matrices do not yet incorporate these populations. This limits the ability of DNAm deconvolution to capture the full complexity of immune cell profiles in PE, where subset-specific responses are crucial to understanding disease progression.[75] In PE, non-classical monocytes contribute to vascular dysfunction [76], macrophages exhibit an imbalance favoring pro-inflammatory M1 phenotypes that impair trophoblast invasion (Reviewed in: [59,77]), and dendritic cells become aberrantly activated near the placenta, exacerbating local inflammation. Although DNAm patterns among these monocyte subsets [76,78] and their derivatives [78–80] are known, they remain underrepresented in current reference matrices. As a result, monocyte counts alone may be insufficient to distinguish between healthy and PE pregnancies; instead, a combination of monocyte count, activation state, and phenotypic changes should be assessed to appropriately estimate the immune dysregulation seen in PE. To address this limitation, future DNAm reference matrices should incorporate subset-specific and activation-state signatures, allowing for more precise characterization of immune dynamics during pregnancy and PE.

### 4.3. Immune dynamics across gestation and the postpartum period

Beyond limitations in deconvolution resolution, it is also important to consider how immune cell profiles shift in response to gestational age, particularly during key windows such as late pregnancy and the postpartum period. For example, monocyte counts are influenced by gestational timing and critical immune transition across pregnancy, contributing to shifts in total cell counts, subset proportions, and differentiation patterns (e.g., macrophages, dendritic cells, T cells), particularly around parturition. In this study, we analyzed GSE37722, a Illumina 27k methylation array extracted from blood samples collected within 24h of delivery, a critical period marked by distinct immune responses.[81] Immune composition is known to change throughout the third trimester and into the postpartum period [59,61], reflecting the dynamic nature of maternal immune adaptation and the transition towards postpartum immune reactivation.

Gestational adaptation of the maternal immune system begins soon after fertilization and continues throughout pregnancy, followed by a return to a pre-pregnancy immune state after delivery and during lactation.[19] To better capture these immune transitions, we applied the Houseman algorithm to two publicly available datasets (GSE37722 and GSE192918) to estimate immune cell composition at various gestational stages. While the role of DNAm in regulating maternal immune physiology during pregnancy and postpartum remain incompletely understood, our findings provide insights into these dynamics. At delivery (GSE37722), genome-wide DNAm profiles in maternal leukocytes showed higher methylation levels in PE cases than in normotensive controls, particularly at promotor CpG sites, suggesting gene repression may contribute to immune dysregulation.[81] Analysis of dataset GSE192918 revealed that 61.63% of CpG sites displayed subtle methylation changes during pregnancy, with more significant shifts occurring postpartum.[82] The second and third highest concentrations of altered CpGs occurred between the first and second trimesters, likely reflecting key stages of embryonic development and organogenesis. Pathway enrichment analyses of GSE192918 highlighted several immune-related pathways, including IL-15 production, Fcγ receptor-mediated phagocytosis in macrophages and monocytes, TREM1 signaling, and IL-7 signaling, suggesting that DNAm changes contribute to shaping the maternal immune landscape.[82] This reshaping was further supported by our cell composition estimates, which showed significant shifts in granulocytes and CD8^+^ T cells between pregnancy and the postpartum period.

In both datasets, the most prominent immune differences were observed between pregnancy (early, mid, at delivery) and the postpartum period. Granulocytes, NK cells, and T cells showed substantial shifts during the transition out of pregnancy. Notably, granulocyte counts, which varied with gestational age, were consistently lower postpartum. These shifts likely reflect the resolution of pregnancy-associated immunotolerance and reactivation of systemic immune responses. Although we initially hypothesized that trimester-specific changes would drive immune cell composition, we observed relative stability across trimesters. This contrasts with earlier reports of gestation-specific immune shifts in pregnancy [19,83,84], emphasizing the complexity of immune regulation at the maternal-fetal interface. In contrast, the postpartum period was marked by significant changes, which may indicate the rebound of systemic immunity following the immunosuppressive state of pregnancy. Although clinical symptoms of PE typically resolve upon delivery of the placenta, inflammation may persist into the mother’s postpartum period (Reviewed In: [57]).

### 4.4. Dataset variability and the importance of clinical metadata

Discrepancies between GSE37722 and GSE192918 could be attributed to differences in sample size, population characteristics, and study design. GSE37722 included only first-time mothers of European descent [81], while GSE192918 included 10 women with uncomplicated pregnancies [82]. Although both studies controlled for key confounding variables such as smoking status, BMI, and age [81,82], other factors such as ethnicity and environmental exposures can still influence DNAm. Additionally, differences in methylation array platforms introduces technical variability that complicates cross-study comparisons. The limited clinical data available for these datasets further limits interpretations, making it difficult to fully understand and contextualize the factors influencing the observed variations in immune profiles. Despite these constraints, both datasets consistently support the idea that immune composition is dynamic across gestation and continues to shift into the postpartum period.

Part of this variability may also reflect the inherent heterogeneity of PE itself, which is clinically classified into early-onset PE (diagnosed at or before 34 weeks of gestation), and late-onset PE (diagnosed after 34 weeks) subtypes.[9,85] These subtypes are associated with distinct pathophysiological features: early-onset PE is commonly linked to placental dysfunction and often presents with intrauterine growth restriction, while late-onset PE is more heterogeneous in origin and may involve maternal cardiovascular or metabolic factors.[86,87] Although immune dysregulation plays a role in both subtypes, its manifestations likely differ.[88] Transcriptional and epigenetic analyses are revealing new molecular phenotypes of PE that may not align directly with traditional clinical classification. Leavey et al. (2018, 2019) identified two additional transcriptional subtypes of PE: canonical and immunological, distinguished by unique gene expression and epigenetic patterns in placental tissue.[89,90] These molecular phenotypes along with observed differences in placental immune cell composition suggest that current clinical definitions may overlook key molecular features driving disease vulnerability and progression.

While DNAm signatures may have the potential to differentiate between early-onset PE, late-onset PE, and healthy pregnancies [89,91–93], our study is limited by the lack of detailed clinical phenotypic data in the datasets analyzed [81,82]. Without specific information on PE subtype or gestational age at diagnosis, we cannot determine whether the observed DNAm changes reflect general PE-associated immune dysregulation or are specific to a particular clinical or molecular subset. Furthermore, the small sample sizes in publicly available datasets like GSE192918 and GSE37722 reduce statistical power to detect subtle changes, especially in heterogeneous conditions like PE.[94] Broader limitations including inconsistent data availability, technical variability across platforms, and publication lag further hinder progress in rapidly evolving fields of epigenetics and immunology. Future studies with comprehensive phenotypic metadata, including detailed information on timing of PE onset, clinical severity, and clinical subtype classification is critical for evaluating the potential of DNAm as a biomarker of PE heterogeneity.[94]

Despite these limitations, our findings suggest that DNAm can be leveraged as a tool to infer immune cell composition and detect immunological activation across pregnancy stages. While most previous studies have focused on fetal-derived tissues or cell-free placenta DNA [47,48,89,90,93,94] in maternal plasma, our study demonstrates that maternal leukocyte DNA offers a valuable and underutilized window into maternal immune adaptations during pregnancy.[81]

### 4.5. Call to action: refining DNAm-based deconvolution for immune research in pregnancy and beyond

Future research should aim for larger, more diverse cohorts and harmonized methodologies to enhance our understanding of the relationship between DNAm, immune cell dynamics, and PE development. A critical component in advancing this field is the development of more robust reference matrices. A limitation in DNAm-based deconvolution remains the lack of pregnancy-specific reference matrices, particularly those that distinguish between monocyte subsets (classical, intermediate, non-classical), macrophage polarization states (M1/M2), and dendritic cell activation markers. Given that many of these immune cell subtypes are critical in our understanding of gestation, and pathologies like PE, refining these reference panels is essential for improving the interpretation of immune changes in pregnancy.

To address this, future DNAm reference matrices should be developed using samples from female individuals, ensuring that sex-specific immune dynamics and hormonal influences on DNAm patterns are accurately captured. Additionally, these reference matrices should be generated using advanced flow cytometry techniques that allow for finer resolution of immune cell subsets and their functional states. Furthermore, menstrual history (date of last menstrual period), pregnancy history (parity), and gestational age should be integrated as key variables in DNAm reference matrices. Primiparous and multiparous individuals exhibit distinct immune adaptations, with prior pregnancies influencing immune tolerance, inflammatory responses, and vascular remodeling.[95] Similarly, menstrual cycles are associated with fluctuations in immune cell composition, particularly in monocytes, neutrophils, and T cells, due to cyclic hormonal changes in estrogen and progesterone levels.[96] These hormonal fluctuations can impact DNAm patterns and immune cell proportions, yet they are not currently accounted for in existing DNAm deconvolution models. Without considering parity and menstrual phase at sample collection, reference matrices may misrepresent immune cell distributions and activation states, leading to confounding effects in pregnancy-related studies.

Beyond pregnancy-specific considerations, the overall health status of donors is a critical factor that should be incorporated into DNAm deconvolution models. Pre-existing conditions such as autoimmune disorders, metabolic syndromes, or chronic inflammation could influence DNAm immune profiles, affecting how they are matched to flow cytometry within reference matrices and ultimately influencing immune cell deconvolution results. Rather than developing reference matrices tailored solely to specific scientific questions, future efforts should prioritize comprehensive, large-scale panels that integrate multiple biological factors including sex, ethnicity, age, and pre-existing health conditions. Ensuring that these key donor metadata are accessible will enhance the accuracy and generalizability of DNAm-based immune profiling.

Reproducibility in DNAm research is heavily dependent on access to raw data, yet many publicly available datasets, including those used in this study, provide only preprocessed methylation data. Without access to raw DNAm intensity values, researchers are limited in their ability to apply alternative normalization methods or validate preprocessing pipelines. This lack of transparency hinders methodological advancements and comparability across studies. To address this, future studies should prioritize the deposition of raw DNAm data alongside processed outputs in public repositories to enable greater reproducibility and flexibility in data analysis. Researchers are encouraged to adhere to the four-step checklist for biological data deposition by Wilson et al. (2021) [97], ensuring that data shared across specialist and general repositories adheres to the FAIR guiding principles—findable, accessible, interpretable, and reusable. This approach will promote consistency, transparency, and broader utility in future studies.

## 5. Conclusion

This study demonstrates the potential of DNAm profiling as a valuable tool for characterizing immune cell dynamics in pregnancy and pregnancy-related disorders. While we did not observe significant differences in DNAm-derived estimated immune cell proportions between preeclamptic and normotensive pregnancies, our analysis revealed significant immune shifts during the transition from pregnancy to the postpartum period. These findings highlight the dynamic nature of maternal immune adaptation and reinforce the importance of longitudinal sampling to fully capture immune changes associated with pregnancy complications. Recent evidence suggests multifactorial model of PE, in which interactions among genetic predisposition, environmental exposures, vascular dysfunction, placental abnormalities, and immune system activation contribute to the onset and distinct clinical presentations of PE. Our study supports this multifactorial view, suggesting that epigenetic changes may both reflect and affect these complex influences. The ability to use DNAm profiles to infer immune cell composition across gestation and into the postpartum period offers promising avenues for non-invasive monitoring and early detection of pregnancy complications. Future studies incorporating longitudinal data that includes pre-PE sampling, gestational, and postpartum timepoints are critical for capturing the full spectrum of immune dynamics and identifying early immune changes preceding clinical onset and understanding the potential long-term effects on maternal and fetal health. However, several limitations must be addressed to strengthen future research in this area. Small sample sizes, technical variability across methylation platforms, and inconsistent or incomplete clinical metadata currently hinder reproducibility and interpretation. Future research with larger, more diverse cohorts and harmonized methodologies will be essential to deepen our understanding of the complex interplay between epigenetic regulation and immune function in PE. In addition, adopting FAIR (findable, accessible, interoperable, reusable) data principles will ensure that future findings are transparent, reproducible, and broadly accessible – ultimately, helping to advance maternal health research on a global scale.

## Supporting information

S1 Fig Principal component analysis (PCA) of GSE37722 and GSE192918. PCA was performed on all probes overlapping the two datasets, the 100 probes used for Houseman deconvolution, and the estimated cell type proportions. Samples are coloured by condition and dataset to visualize batch effects.
(TIFF)
