# Supplementary figures and images for: Distinct DNA methylation signatures in maternal blood reveal unique immune cell shifts in preeclampsia and the pregnancy-postpartum transition
Source: PLoS One. 2026 Feb 25;21(2):e0343041. doi: 10.1371/journal.pone.0343041 (PMC12935243; doi:10.1371/journal.pone.0343041)

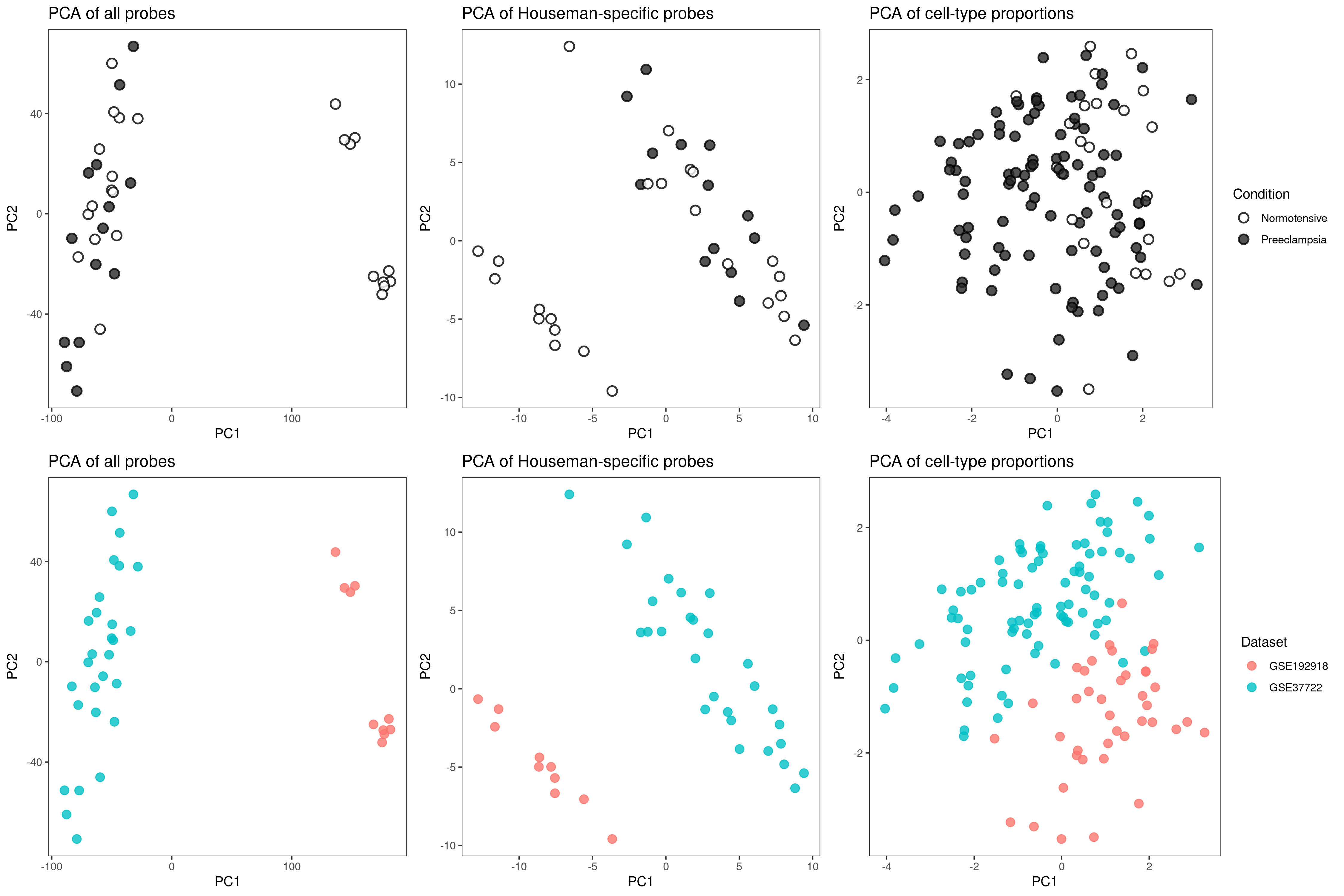

Supplement: S1 Fig — PCA was performed on all probes overlapping the two datasets, the 100 probes used for Houseman deconvolution, and the estimated cell type proportions. Samples are coloured by condition and dataset to visualize batch effects. (TIFF) [file pone.0343041.s001.tiff]
